# In-Vitro Endothelialization Assessment of Heparinized Bovine Pericardial Scaffold for Cardiovascular Application

**DOI:** 10.3390/polym14112156

**Published:** 2022-05-26

**Authors:** My Thi Ngoc Nguyen, Ha Le Bao Tran

**Affiliations:** 1 Laboratory of Tissue Engineering and Biomedical Materials, University of Science, Ho Chi Minh City 700000, Vietnam; ntnmy@hcmus.edu.vn; 2 Department of Physiology and Animal Biotechnology, Faculty of Biology—Biotechnology, University of Science, Ho Chi Minh City 700000, Vietnam; 3 Vietnam National University, Ho Chi Minh City 700000, Vietnam

**Keywords:** heparinize, bovine pericardium, decellularization, scaffold, hemocompatibility, endothelialization

## Abstract

(1) Background: Hemocompatibility is a critical challenge for tissue-derived biomaterial when directly contacting the bloodstream. In addition to surface modification with heparin, endothelialization of the grafted material is suggested to improve long-term clinical efficacy. This study aimed to evaluate the ability to endothelialize in vitro of heparinized bovine pericardial scaffolds. (2) Methods: bovine pericardial scaffolds were fabricated and heparinized using a layer-by-layer assembly technique. The heparinized scaffolds were characterized for heparin content, surface morphology, and blood compatibility. Liquid extraction of the samples was prepared for cytotoxicity testing on human endothelial cells. The in-vitro endothelialization was determined via human endothelial cell attachment and proliferation on the scaffold. (3) Results: The heparinized bovine pericardial scaffold exhibited a heparin coating within its microfiber network. The scaffold surface immobilized with heparin performed good anti-thrombosis and prevented platelet adherence. The proper cytotoxicity impact was observed for a freshly used heparinized sample. After 24 h washing in PBS 1X, the cell compatibility of the heparinized scaffolds was improved. In-vitro examination results exhibited human endothelial cell attachment and proliferation for 7 days of culture. (4) Conclusions: Our in-vitro analysis provided evidence for the scaffold’s ability to support endothelialization, which benefits long-term thromboresistance.

## 1. Introduction

Bovine pericardium, first utilized for bioprosthetic heart valve fabrication, has been widely used in clinical practice [1,2]. Decellularization is considered the critical factor that significantly promotes bovine pericardium and other tissue-derived extracellular matrix (ECM) for biomedical research and application. This technique focuses on eliminating xenogenic antigenicity while preserving the native ECM structural, mechanical, and biologic properties. For long-term stability in vivo, further fixation or crosslinking, i.e., glutaraldehyde treatment, was adopted to prepare tissue ECM-derived biomaterials. Decellularized and glutaraldehyde treated bovine pericardium are now commonly used as commercially available products proven for their durability, biocompatibility, and an appropriate effect on host tissue remodeling. In the field of cardiovascular practice, it has been found that bovine pericardial patches revealed significantly less suture line bleeding (at 14%) than a prosthetic patch material (at 55% in the Dacron patches) [3,4]. Somehow, there is still a demand for a non-thrombogenic strategy due to the particular risk of coagulation on this biomaterial when directly contacting the bloodstream [4,5,6,7].

To prevent early thrombosis after implantation, patients are treated with anticoagulation drugs via injection or oral use. Another potential solution is to improve the hemocompatibility of blood-contacting biomaterials. Material-surface modification has been taken into account, including immobilization of bioactive anticoagulants. Heparin is the most commonly used in systemic anticoagulant therapy. Heparin belongs to the family of glycosaminoglycans and is characterized as a highly sulfated linear polysaccharide, with large quantities of hydroxyl, amino, carboxyl, and sulfonyl. Typically, heparin is linked to the extracellular matrix (ECM) using an active crosslinker such as glutaraldehyde and (1-(3-dimethylaminopropyl)-3-ethylcarbodiimide hydrochloride) (so-called EDC) [5,6,7]. These chemical immobilization methods have shown effectiveness in grafting heparin onto ECM materials with considerable stability, whereas the residual amount of crosslink reagent presents potential cytotoxicity and calcification. In addition, a heparin incorporation method by layer-by-layer (LBL) assembly was reported, in which complex multilayers of iron-heparin were deposited onto pericardial ECM fibers [8,9]. This procedure effectively reduced blood-clot formation on the material surface. It also revealed heparin release and maintained an antithrombotic effect during a 30-day washing in phosphate buffer saline [9]. However, when reaching the end-point of heparin release, thrombus formation would likely occur, which could diminish the clinical efficacy of this material. Thus, there is a demand for a long-term strategy to improve the hemocompatibility of this material.

Indeed, the principal player in thrombosis prevention is the endothelial layer on blood-contacting surfaces. The endothelial layer was shown to not occur on any cardiovascular device immediately after implantation, causing thrombosis [10]. For early endothelialization, biochemical modification of the surfaces and immobilization of biomolecules to the surfaces have been useful. The present publications have provided evidence for the attachment of endothelial cells on heparin-modified material surfaces [11,12,13]. At the same time, pericardial scaffolds were also proven to support endothelial cell adherence and proliferation [14]. Therefore, it could be predicted that a heparinized bovine pericardial scaffold might exhibit a positive endothelialization effect. Our previous study successfully incorporated heparin onto a bovine pericardial scaffold, which indicated good thrombosis resistance. In the current research, we aimed to investigate further the ability of the scaffold to support endothelialization in vitro. The heparinized bovine pericardial scaffolds were examined for their cytotoxicity and effect on human endothelial cell attachment and proliferation.

## 2. Materials and Methods

### 2.1. Preparation of Bovine Pericardial Scaffold with Heparin Modification

Bovine pericardial scaffold (BPS) was prepared and heparinized according to our previous publications [9,15]. Briefly, the bovine pericardium was decellularized with 10 mM Tris-HCl (Sigma Aldrich, St. Louis, MI, USA) and 0.15% SDS (Sigma Aldrich, USA), followed by further stabilizing in 0.1% glutaraldehyde (Sigma Aldrich, USA). The BPS was obtained after lyophilization and sterilization by gamma irradiation. The BPS underwent a heparin modification, in which the BPS was incubated in DHI ([Fe(OH)_2_]^+^) (Sigma Aldrich, USA) and heparin solution (Sigma Aldrich, USA). The procedure includes seven cycles of layer-by-layer (LbL) assembly for heparin deposition on the scaffold. In each cycle, BPS was immersed in DHI solution for 5 min, then washed in 0.9% NaCl (Sigma Aldrich, USA) solution for 5 min, followed by heparin condition for 5 min at room temperature. The heparinized BPS (HepBPS) were examined freshly after fabrication. 

### 2.2. Toluidine Blue O (TBO) Assay

Heparin content in HepBPS was measured by TBO assay, which colorimetrically measures glucosamine concentration. HepBPS samples were lyophilized and cut into 1 × 1 cm and incubated in 0.04% TBO solution (Sigma-Aldrich, USA) for 4 h. The samples were washed in deionized water to eliminate unreacted TBO solution and immersed in 80% Ethanol/0.1 M NaOH mixture to extract the heparin/TBO complex. The finalized solution was measured for absorbance at 530 nm using a microplate reader (EZ Read 400, Biochrom, Cambridge, UK). Heparin content on samples was evaluated by comparing with a calibration curve. 

### 2.3. Structural Characteristics

#### 2.3.1. Hematoxyline and Eosin Staining 

The BPS and HepBPS were fixed with 10% formalin, embedded in paraffin, and processed for staining with hematoxylin and eosin (H&E). Images were taken using Olympus CKX-RCD microscope (Tokyo, Japan) equipped with a DP2-BSW microscope digital camera. 

#### 2.3.2. Scanning Electron Microscopy

The BPS and HepBPS were washed once by PBS 1X and fixed in a homemade fixation solution (1.35% Paraformaldehyde, 3% Glutaraldehyde, 4.45% Succrose in PBS 1X, pH 7.4) for SEM evaluation. All samples were fixed under 4 °C for 24 h and dehydrated through a graded series of ethanol. Samples were sputter coated with a layer of gold for 60 s to observe the high-resolution images. The ultrastructure of all samples was investigated using scanning electron microscopy (SEM, JSM-6510, JEOL, Tokyo, Japan).

### 2.4. Thrombosis and Platelet Attachment

To evaluate the potential antithrombogenicity, the scaffolds were immersed in citrated whole blood. After incubation at 37 °C for 2 h, blood coagulation was activated by adding with 0.1 M CaCl_2_ solution (ration 1:10, *v*/*v*) [9]. The scaffolds were briefly flushed with distilled water and examined for the presence of blood clots on the surface. 

In platelet attachment test, platelet rich plasma (PRP) was collected from whole blood. Each scaffold was incubated with PRP at 37 °C for 2 h, followed by washing with PBS 1X. The attachment of platelets on the scaffolds was examined by SEM.

### 2.5. In-Vitro Cytotoxicity Tests

#### In-Vitro Cell Culture 

HUVEC were purchased from the American Type Culture Collection (ATCC, Manassas, VA, USA). HUVEC were cultured in vascular cell basal medium (ATCC, USA) supplemented with Endothelial Cell Growth Kit-VEGF and Penicillin-Streptomycin-Amphotericin B Solution (ATCC, USA) (so-called complete culture medium). The cells were cultured at 37 °C and 5% CO_2_, with a constant humidity of 95% (Panasonic, Osaka, Japan). Culture medium was freshly changed every 2 days. HUVEC at 4th passage were used for the assessment. HUVEC suspension was prepared by trypsinization and seeded into each well of a 96-well plate at 1 × 10^4^ cells/mL. The culture plate was incubated in 5% CO_2_ humid atmosphere at 37 °C overnight for cell attachment and spreading.

### 2.6. Sample Extraction and Testing

The HepBPS samples underwent either non-wash or 24 h wash in PBS 1X solution before the extraction process. The extract of the samples was prepared following the ISO 10993-12 standard. Complete culture medium was used as an extraction medium. Each sample was extracted in the extraction medium (1 cm^2^ per 1 mL) at 37 °C for 24 h to obtain a test liquid extract. The culture medium was removed completely on the test day and the test extract (100 μL) was added. Culture medium and culture medium containing 20% DMSO were used as the blank and positive control, respectively. The plate was incubated in 5% CO_2_ humid atmosphere at 37 °C for further 24 h. Cell viability was determined using MTT assay. 

### 2.7. Cytotoxicity Determination

After 24 h incubation, tested liquid extract and controls were removed, followed by adding 100 µL of salt (MTT) solution (Sigma-Aldrich, St. Louis, MI, USA) (0.5 mg/mL) into each well. The resultant mixture was incubated in 5% CO_2_ humid atmosphere at 37 °C for 4 h for the formation of formazan crystals. The MTT solution was removed and stopping solution of DMSO and Ethanol (Sigma-Aldrich, USA) (ratio 1:1, *v*/*v*) was added to the wells. Stopping solution was mixed in wells to completely dissolve the formazan crystal. The optical density (OD) was measured at 570 nm with the ELISA Microplate Reader (Biochrom EZ Read 400, Cambridge, UK).

Sample cytotoxicity was determined according to the relative growth rate (RGR, %). Formular for RGR of test sample (%) was as (OD_test_/OD_blank_) × 100%. The liquid extract is confirmed as no-toxic to the cells if the RGR value is higher than 70 %, according to ISO 10993-5 guidance [2]. 

### 2.8. Cell Attachment on the Scaffolds 

To examine cell attachment, the HepBPS was cut into round specimens and placed into the wells of 96-well plate. HUVEC were seeded into each well at a density of 3 × 104 cells per well and cultured for 24 h. The specimen seeded with HUVEC were collected, rinsed once in PBS 1X, and incubated in Calcein A solution (Thermo Scientific, Waltham, MA, USA) (ration 1:1000 in PBS, *v*/*v*) for 35 min at 37 °C. Samples were briefly rinsed in PBS and imaged using a fluorescence microscope (Olympus IX81, Olympus, Tokyo, Japan). HUVEC adherence and morphology were also observed by scanning electron microscope as per earlier described procedure. 

### 2.9. Cell Proliferation Assay 

For the proliferation evaluation, HUVEC were seeded into the well containing HepBPS specimen with a density of 3 × 10^4^ cells. HUVEC proliferation was examined by a Cell Counting Kit-8 (CCK-8) (Sigma-Aldrich, USA) on day 1, 4, and 7. At each time point, medium containing 10% CCK-8 was added to each well and incubated for 4 h at 37 °C. The medium was transferred into a new 96-well plate for measuring the optical absorbance at 450 nm with ELISA Microplate Reader.

### 2.10. Statistical Analysis

All data sets were analyzed by Student’s *t*-test for comparison between two groups using the GraphPad 8.0 software. Data were expressed as mean ± standard division of the mean (SD), and statistical significance was set at *p* < 0.05. All experiments were conducted in triplicate.

## 3. Results

### 3.1. Characterization of the Heparinized Bovine Pericardial Scaffold (HepBPS)

**Figure 1:**
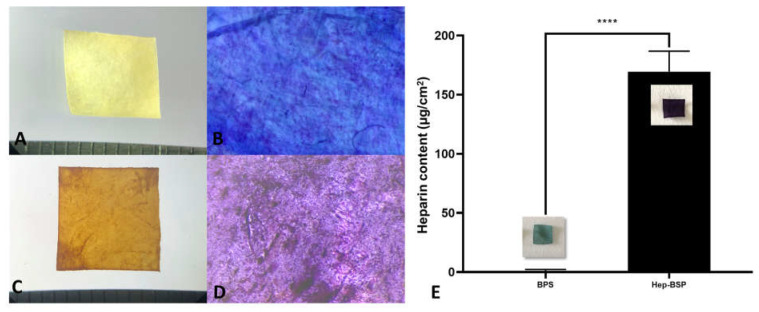
Preparation of heparinized bovine pericardial scaffold. (**A**)—Macroscopic observation of Bovine pericardial scaffold (BPS). (**B**)—Stereo microscope image of BPS after toluidine blue incubation. (**C**)—Heparinized bovine pericardial scaffold (HepBPS). (**D**)—Stereo microscope image of HepBPS after toluidine blue incubation. (**E**)—Heparin quantification of the scaffolds. ****: *p* value < 0.0001.

Macroscopical observation presented a significant difference between the BPS (Figure 1A) and HepBPS (Figure 1C), in which HepBPS adopted a diffuse brown appearance. After incubation with the TBO solution, the BPS sample obtained a light blue color (Figure 1B), whereas a homogeneous presence of purple crystals was clearly detected on the HepBPS sample (Figure 1D). This result indicated a successful deposition of heparin on the scaffold using the LbL assemble technique. Quantitatively, heparin content in the HepPBS was determined as 169.5 ± 17.31 mg/cm^2^ (Figure 1E).

**Figure 2:**
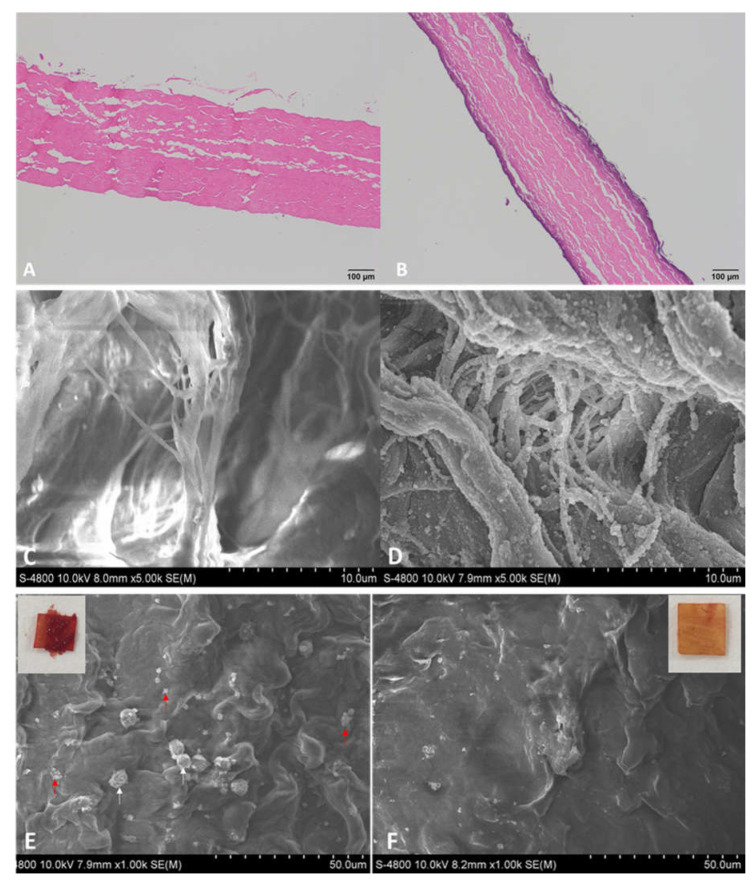
Characterization of heparinized bovine pericardial scaffold. (**A**)—Hematoxylin and Eosin (H&E) staining of Bovine pericardial scaffold (BPS). (**B**)—H&E staining of Heparinized bovine pericardial scaffold (HepPBS). (**C**)—Scanning electron microscope (SEM) of BPS. (**D**)—SEM of HepPBS. (**E**)—Platelet attachment and SEM examination of BPS. (**F**)—Platelet attachment and SEM examination of HepBPS. H&E staining: All scale bars are 10 μm. SEM: All scale bars are 50 μm. Red arrows indicate platelets. White arrows indicate white blood cells.

The pericardium derived scaffold was composed of extracellular matrix fibers, which are basic and was stained with eosin as an acid dye (Figure 2A). After heparin modification, the outer layer of sections was stained dark blue (Figure 2B). Heparin is highly acidic because of sulfate and carboxylic acid groups [16,17], which could generate selective reactions with hematoxylin as a basic dye. SEM images confirmed this variation in surface morphology between these surfaces before and after heparin modification (Figure 2C,D). After modification with heparin by the LbL technique, SEM illustration showed the deposition of the DHI/heparin complex as coatings around the fibrils of the HepBPS structure (Figure 2D).

There was the apparent formation of a blood clot on the surface of the BPS sample compared with the thrombus-free surface of HepBPS sample. On the BPS membrane, some adhered platelets were found (Figure 2E), which corresponded to its thrombus formation. HepBPS performed better hemocompatibility, with nearly no platelets attached (Figure 2F).

### 3.2. In-Vitro Cytotoxicity Tests

**Figure 3:**
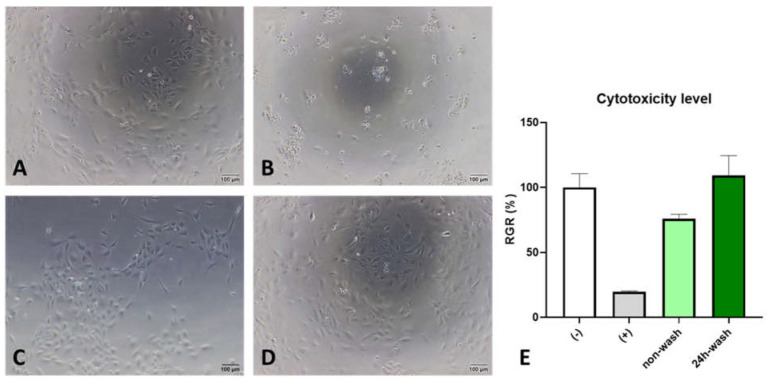
Observation of HUVEC cultured in different solutions. (**A**)—Culture medium as a negative control. (**B**)—Liquid extract from latex as a positive control. (**C**)—Liquid extract from a non-washed HepBPS. (**D**)—Liquid extract from HepBPS after 24 h wash in PBS 1X. (**E**) Cytotoxicity determination (Magnification 10×). All scale bars are 100 µm.

In-vitro cytotoxicity tests aimed to provide predictive evidence of biocompatibility. HUVEC were cultured in the condition of the HepBPS liquid extract. After 24 h incubation, no cell death and changes in cell pattern were observed in the complete medium as a negative control (Figure 3A). Meanwhile, 20% DMSO solution as a positive control was highly toxic to the cells, resulting in cell death and detachment (Figure 3B). For the non-washed sample, which imitated the immediate effect of HepBPS when implanted, there was a proper negative effect on cell viability indicated by the decrease in cell adherence density (Figure 3C) and relatively low RGR percentage (75.83%). After 24 h washing in PBS 1X, the cytotoxicity of the HepBPS liquid extract was pretty low, showing as high a level of RGR as 96.56% (Figure 3E). The liquid extract of DMSO caused harsh affects on cell viability, with RGR values at 3%.

### 3.3. Cell Attachment on the Scaffolds

**Figure 4:**
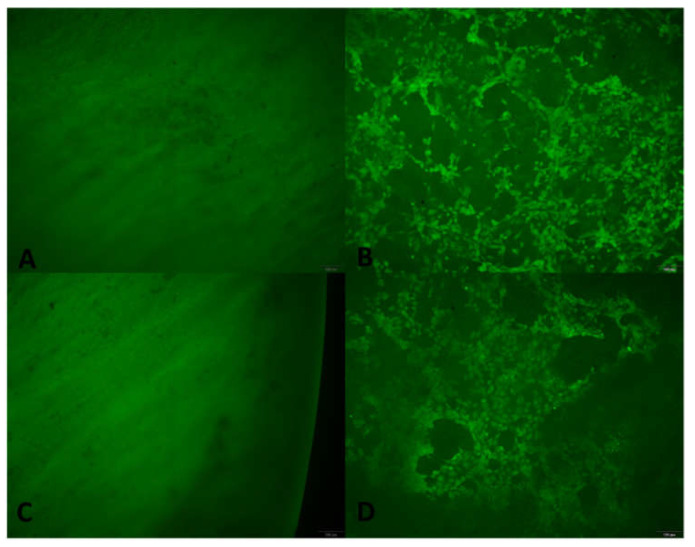
Calcein staining of BPS and HepBPS after HUVEC seeding. (**A**)—BPS without cell seeding. (**B**)—BPS seeded with HUVEC. (**C**)—HepBPS without cell seeding. (**D**)—HepBPS seeded with HUVEC. All scale bars represent 100 μm.

**Figure 5:**
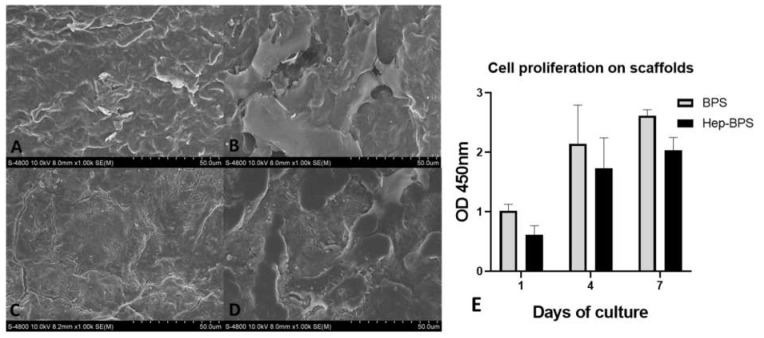
Assessment of cell attachment by SEM and cell proliferation on scaffolds. (**A**)—BPS without cell seeding. (**B**)—BPS seeded with HUVEC. (**C**)—HepBPS without cell seeding. (**D**)—HepBPS seeded with HUVEC. (**E**)—CCK8 assay for cell proliferation. All scale bars represent 50 μm.

HUVEC were used to examine the cell attachment support of the scaffolds. HUVEC were seeded onto either BPS or HepBPS and visualized by calcein staining (Figure 4) and SEM (Figure 5). HUVEC attachment on both scaffolds was detected after culturing for 24 h. Somehow, the density of endothelial cells on the HepBPS membrane was similar to that on the surface of the BPS membrane (Figure 4B,D). SEM images also exhibited HUVEC spreading morphology with cellular projections interacting with scaffold components.

### 3.4. Cell Proliferation on the Scaffolds

Cell proliferation was determined by CCK8 assay at different time points, on day 1, 4, and 7, as shown in the chart (Figure 5E). In both types of scaffolds, the proliferation rate of HUVEC within the first four days was recorded. During the next 4 and 7 days, the optical absorbance of the incubated solution did not significantly increase, indicating a limited growth rate after 7 days. However, data indicated a slower cell growth rate in the scaffolds after heparinization, which was shown as a significantly higher in OD value of the BPS group compared to the HepBPS group.

## 4. Discussion

Acellular-tissue-matrix-derived biomaterials provide extensive applications in clinical surgery due to their ready availability and potential tissue regeneration. In the case of cardiovascular patch fabrication, bovine pericardium is the most well-known material for its suitable thickness, low rate of suture bleeding, good biocompatibility, and mechanical properties. As the cardiovascular patch is always in direct contact with the bloodstream, besides the mentioned advantages, this type of material still has a drawback regarding its hemocompatibility. A hemocompatible material should not cause any adverse interactions with blood components, especially not activating blood coagulation or blood clots. However, the pericardium is a biologic tissue; it is mainly composed of collagen fibers, which create an attractive surface for the absorption of plasma proteins and platelet attachment. Therefore, blood clots or thrombosis on the material surface is an obvious consequence. Therefore, there is a demand to improve the hemocompatibility of this material.

To prevent early thrombosis after implantation, surface modification strategies have been developed to improve blood compatibility, including the immobilization of bioactive anticoagulants. In this case, heparin is the most commonly used in systemic anticoagulant therapy. Our previous study successfully established a heparinization of the bovine pericardial scaffold using the layer-by-layer assembly technique [9]. The results showed that heparin could be incorporated into the bovine pericardial scaffold, as proven via SEM images, histological analysis, and heparin amount assay. The seven cycles selected for the LbL technique was determined due to the high level of heparin accumulation, which indicated seven assembly cycles as the heparin immobilization threshold.

In this current study, we also used this technique to immobilize heparin to the scaffolds and further tested for their anti-thrombotic activity and endothelialization support. The results confirmed that the preparation technique effectively deposited heparin in the bovine pericardial scaffold. The macroscopic analysis demonstrated adequate uniform coverage over the surface of the scaffold. Compared with the untreated scaffolds, heparinization resulted in a visually clot-free surface and empty platelet attachment. This observation was similar to the present studies on adopting heparin for surface modification, including decellularized vascular graft and live matrix [8,9,18,19]. Accordingly, surface modification with heparin could provide anti-thrombosis with two effects. In a direct impact, a surface with heparin would prevent the adsorption of plasma proteins and platelet adherence, therefore successfully creating an anti-thrombosis surface. In the other way, after modification, the material can release heparin which, in turn, suppresses the activity of thrombin, thus creating an anti-thrombosis microenviroment within the material and keeping the material surface in a free-clotting condition [9,18]. 

Endothelial cells form a consensus layer in the blood vessel and maintain the normal physiology condition of blood vessel. One of the most essential functions of the endothelial cell is to prevent thrombosis. Therefore, from a material perspective, endothelialization is a process in which the endothelial cells can form a layer on the material surface. If the cardiovascular patch could achieve this ideal stage, long-term anti-thrombosis could be guaranteed. Our published data showed that the heparinized bovine pericardial scaffolds could release heparin and maintain the anti-thrombus stage for 30 days [9]. This result indicated that the blood clotting on the scaffold surface possibly happens as a consequence of complete heparin release. Therefore, the ability to support endothelialization could, in turn, ensure the blood compatibility of the scaffold. Overall, our study demonstrated that HepBPS could provide an appropriate attachment and proliferation of human endothelial cells. However, before this performance, the HepBPS should undergo 24 h washing in PBS 1X solution; otherwise, potential cytotoxicity could be a certain (as shown in cytotoxicity assay, Figure 3). Although heparin is frequently used as an anticoagulant, there were findings of the toxic effects of heparin in cell cultures [20]. Additionally, there was the presence of DHI ions in the HepBPS, which was also unloaded during incubation and possibly caused a decrease in cell viability. Meanwhile, the in-vitro cytotoxicity of the releasing heparin and DHI ions in our specific case and present publications [9,10,20] remains unsolved, thus demanding a detailed investigation in further study. Endothelialization was detected on HepBPS via endothelial cell attachment and proliferation on the scaffold (as shown in Figure 4 and Figure 5). This implied the two-phase anti-thrombotic activity of the HepBPS when implanted. In the early time of implantation, heparin release and heparin on the graft surface would take the main role in anti-thrombus. Then, the scaffold itself could support endothelialization, which benefits the long-term thromboresistance by preventing the sub-endothelial matrix from blood contact. 

As mentioned earlier, endothelial cells were confirmed to attach to a heparin-modified material surface [11,12,13,17], which was also found in our current result. The results also showed that the endothelialization effect on the heparinized scaffolds was not comparable to the un-modified one, which might raise a contradiction on the initial purpose of employing heparin on the bovine pericardial scaffolds. In fact, SEM images revealed completely coated microfibers with the heparin/DHI complex after the LbL assembly procedure. Consequently, cell attachment would be dismissed due to reduced or a lack of cell–matrix interaction. Endothelial cells were shown not to express direct receptors for heparin. The positive effects of the heparin immobilized scaffold on endothelial cells were described via “bridging” molecules, e.g., chitosan, gelatin [11,17], or binding and stabilizing cell growth factors, e.g., VEGF [12,13]. Therefore, the decrease in cell adherence or proliferation on HepBPS could be explainable. Another mechanism for endothelialization relates to the protein adsorption level on the scaffold. In-vivo transplantation clearly demonstrates that protein adsorption is the first event that happens at the interface, leading to a later material associated with cell attachment and growth [11,13]. The heparinized scaffold is negatively charged, possibly minimizing negatively charged protein adsorption [18,21] and endothelialization. Taken together, our in-vitro results on HepBPS remained the predictive evidence for the scaffold’s ability to support endothelial cell attachment and proliferation in both cases, including the later period of heparin release in vitro/in vivo, long-term interaction, and uptaking of plasma proteins and growth factors in vivo. Therefore, the mechanisms for interactions between the bovine pericardium, heparin and cells need to be performed. Further investigations on these undefined behaviors of the heparinized bovine pericardial scaffold in vitro and in vivo are also recommended to provide a better understanding of the clinical application. 

## 5. Conclusions

In this study, heparinized bovine pericardial scaffolds were constructed via LbL assembly successfully. The results demonstrated that the use of the heparinization technique provided anti-thrombosis and prevented platelet adhesion. After an adequate heparin release by immersing in PBS 1X, the scaffolds could support the attachment and proliferation of endothelial cells, which exhibited a promising effect on endothelialization and long-term hemocompatibility. However, in order to achieve complete understanding of its behaviors, additional investigation should be performed.

## Data Availability

Data presented in this study are available in the article.
